# Lipoic Acid Based Redox‐Responsive Degradable Antimicrobial Polymers

**DOI:** 10.1002/marc.202500224

**Published:** 2025-06-17

**Authors:** Anupama Giri, Md Aquib, Anmol Choudhury, Vinod Kumar Kannaujiya, Jie Lay Lim, Zi Gu, Megan D. Lenardon, Cyrille Boyer

**Affiliations:** ^1^ Cluster for Advanced Macromolecular Design (CAMD) and School of Chemical Engineering UNSW Sydney New South Wales Australia; ^2^ School of Biotechnology and Biomolecular Sciences UNSW Sydney New South Wales Australia

**Keywords:** antimicrobial polymers, degradable polymers, lipoic acid, self‐immolative polymers

## Abstract

The rise of multidrug‐resistant (MDR) pathogens poses a critical threat to global health, exacerbated by the overuse of antibiotics and the lack of effective alternatives. Antimicrobial peptides (AMPs) have emerged as promising candidates due to their broad‐spectrum activity and unique mechanisms of action. However, several challenges such as enzymatic degradation, high production costs, and potential cytotoxicity have hindered their clinical translation. To overcome these limitations, antimicrobial polymers (APs) inspired by AMPs have been developed using controlled/living polymerization techniques. In this study, a series of degradable, disulfide‐containing antimicrobial polymers incorporating benzyl lipoate, a lipoic acid (LA) derivative, is synthesized via reversible addition‐fragmentation chain transfer (RAFT) polymerization. Benzyl lipoate is prepared by modification of LA with benzyl alcohol to introduce a hydrophobic moiety and copolymerized with a primary amine‐containing cationic monomer and hydrophilic co‐monomers, including hydroxyethyl acrylamide (HEAm) and poly(ethylene glycol) methyl ether acrylate (PEGMEA). The resulting polymers demonstrated antimicrobial activity against drug‐resistant *Pseudomonas aeruginosa*, improved hemocompatibility, and redox‐responsive degradability. This study highlights the potential of disulfide‐based APs as a next‐generation strategy for combating MDR infections while ensuring controlled degradability.

## Graphical abstract

Antimicrobial polymers are emerging as a vital tool against drug‐resistant pathogens. This article introduces a new class of degradable, disulfide‐containing polymers synthesized through reversible addition‐fragmentation chain transfer (RAFT) polymerization. These innovative materials, featuring benzyl lipoate, effectively combat *Pseudomonas aeruginosa* while demonstrating enhanced hemocompatibility and redox‐responsive degradation. This approach represents a promising next‐generation strategy for tackling multidrug‐resistant infections with precisely controlled biodegradability.

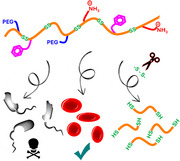

## 1 Introduction

The recent COVID‐19 pandemic has underscored humanity's ongoing susceptibility to infectious diseases [1]. Although the recent pandemic was caused by a virus, infections from pathogenic bacteria and fungi pose an equally serious threat due to the rise of multidrug resistance (MDR) pathogens [2, 3]. This alarming health crisis, driven by the overuse and misuse of antibiotics [4, 5], has resulted in the annual consumption of more than 100 000 tons of these drugs across humans and livestock [6]. In 2019, MDR infections were estimated to be directly responsible for 1.27 million deaths globally and to have contributed to an additional 4.95 million fatalities [7]. Recognizing the severity of the issue, the World Health Organization (WHO) has identified antimicrobial resistance (AMR) as one of the most pressing global health concerns [8]. Beyond its impact on human health, AMR also threaten animal health in the agricultural sector, underscoring the urgent need for innovative solutions for food security.

Antimicrobial peptides (AMPs) are essential components of the innate immune systems in multicellular organisms, serving as a primary defence against microbial infections [9, 10]. Thus, they have attracted considerable interest as promising candidates for new antimicrobial agents [11, 12]. Typically composed of 10–50 amino acids with both hydrophobic and cationic groups, AMPs exhibit broad‐spectrum antimicrobial activity [13, 14, 15]. Their cationic groups strongly interact with negatively charged microbial membranes [16, 17, 18]. In addition, their hydrophobic groups interact with the microbial membrane, leading to structural damages and eventual cell death [16]. This lytic mechanism is believed to make AMPs less susceptible to resistance development [19, 20, 21]. However, their clinical translation faces several challenges, including limited pharmacokinetic stability, enzymatic degradation, and potential cytotoxicity [22, 23, 24]. Furthermore, their large‐scale production is hindered by complex, labour‐intensive synthesis procedures, and high manufacturing costs [25, 26].

To address these challenges, researchers are taking inspiration from natural peptides and leveraging on advanced polymerization techniques to enhance the synthesis and performance of antimicrobial polymers (APs) [27, 28, 29, 30]. A key approach involves reversible‐degeneration radical polymerization, which provides a cost‐effective and scalable method while enabling precise control over degree of polymerization, composition, and polymer architecture [31, 32, 33, 34, 35, 36, 37]. Traditional cationic antimicrobial polymers primarily consist of cationic and hydrophobic moieties. However, the strategic incorporation of hydrophilic functionalities has emerged as a promising strategy, not only to enhance hemocompatibility and improve aqueous solubility but also to modulate their antibacterial mechanisms [38]. Indeed, such structural modifications can lead to interactions with bacteria that differ significantly from those of conventional cationic polymers [39, 40, 41]. For instance, while traditional polymers often rely on generalized membrane disruption driven by broad electrostatic and hydrophobic effects, the refined amphiphilic balance or inclusion of specific responsive units in these terpolymers APs may enable more selective membrane targeting, altered modes of membrane permeabilization, or even engagement with intracellular components, thereby providing distinct mechanistic pathways that offer valuable insights.

Various studies [16, 42–54], including those from our group, have investigated how amphiphilic balance, hydrophobic group, degree of polymerization, and polymer topology influence their antimicrobial activity and biocompatibility. However, concerns persist regarding the long‐term accumulation of these synthetic materials in biological systems [55, 56]. To mitigate this, researchers have developed stimuli‐responsive self‐immolative polymers, designed to degrade in response to specific internal or external triggers such as pH, light, redox or enzymatic activity [57, 58, 59, 60, 61, 62, 63, 64]. For instance, various self‐immolative polymer backbones have been constructed from materials including polyesters [65], polyurethanes [66], polycarbonates [67, 68, 69], polysulfides [70, 71], polyglyoxylates [72, 73], and polyphthalaldehydes [74, 75]. These polymers degrade into their monomeric components upon removal of their end‐caps, making them highly promising for biomedical applications [60, 76–79]. Among these degradable systems, lipoic acid (LA), an abundant, naturally occurring disulfide‐containing compound [80], presents a unique strategy for designing antimicrobial polymers with controlled degradation [81]. The dynamic disulfide bonds in poly(LA) enable responsive degradation under reductive conditions, particularly in intracellular environments with elevated glutathione concentrations [82, 83]. This characteristic makes LA‐based polymers highly attractive for clinical applications, as controlled degradation facilitates polymer clearance and minimizes potential cytotoxicity [83, 84, 85, 86]. However, only a few studies have explored the use of LA in antimicrobial applications. For example, the conjugation of LA with antimicrobial peptides have been shown to enhance antimicrobial activity against *Staphylococcus aureus* (SA) and methicillin resistant *S. aureus* (MRSA) by increasing membrane depolarization and permeability [87]. In a more recent example, cationic, cyclic oligo(disulfide)s derived from lipoic acid have demonstrated strong potential as a novel antimicrobial agent for MRSA and wound infections [86], further highlighting their promise as next‐generation antimicrobial materials [87, 88, 89, 90, 91, 92].

In this work, we investigated the incorporation of a LA derivate, benzyl lipoate (BL), into synthetic APs. BL was copolymerized with a primary amine‐containing cationic monomer and hydrophilic comonomers, including hydroxyethyl acrylamide (HEAm) and poly(ethylene glycol) methyl ether acrylate (PEGMEA), by reversible addition fragmentation chain transfer (RAFT) polymerization technique. Building upon our prior investigations into APs design [46], we synthesized a series of APs incorporating degradable, disulfide‐containing LA‐based fragments into the polymer backbone. We explored the effect of the polymer compositions and hydrophilic groups on the antibacterial activity against four Gram‐negative and one Gram‐positive bacterial strains, as well as their hemocompatibility with red blood cells and cytotoxicity in 3T3 fibroblast cells. The resulting polymers, comprising BL and PEGMEA, demonstrated enhanced antimicrobial activity and improved hemocompatibility, while undergoing redox‐responsive degradation in the presence of a reducing agent. This research advances the development of degradable disulfide‐based antimicrobial materials, offering a promising strategy for tackling microbial infections.

## 2 Results and Discussion

### 2.1 Lipoic Acid‐Based Polymer Design and Synthesis

Our previous work, along with other studies on APs, has explored key parameters, including amphiphilicity, degree of polymerization (DP), polymer architecture, and molecular weight, on antimicrobial activity and biocompatibility [16, 43, 93]. Our findings indicated that an optimal DP of 20–50 with a cationic charge content of ≈50% results in high antimicrobial efficacy while preserving biocompatibility [42, 43, 46]. In this study, we investigated the incorporation of BL, a LA derivate, into APs and its impact on the antimicrobial activity. LA was first modified with benzyl alcohol to produce BL (Figures S1 and S2), which serves as the hydrophobic monomer and introduces degradable disulfide groups into the polymer backbone. The successful synthesis of BL using EDC/DMAP coupling was confirmed by ¹H NMR spectroscopy by the disappearance of the benzylic protons (‐CH_2_‐) signal at 4.5 ppm from benzyl alcohol and the presence of a new peak at 5.10 ppm attributed to benzylic protons in adjacent of the ester group (Figure S1).

**FIGURE 1:**
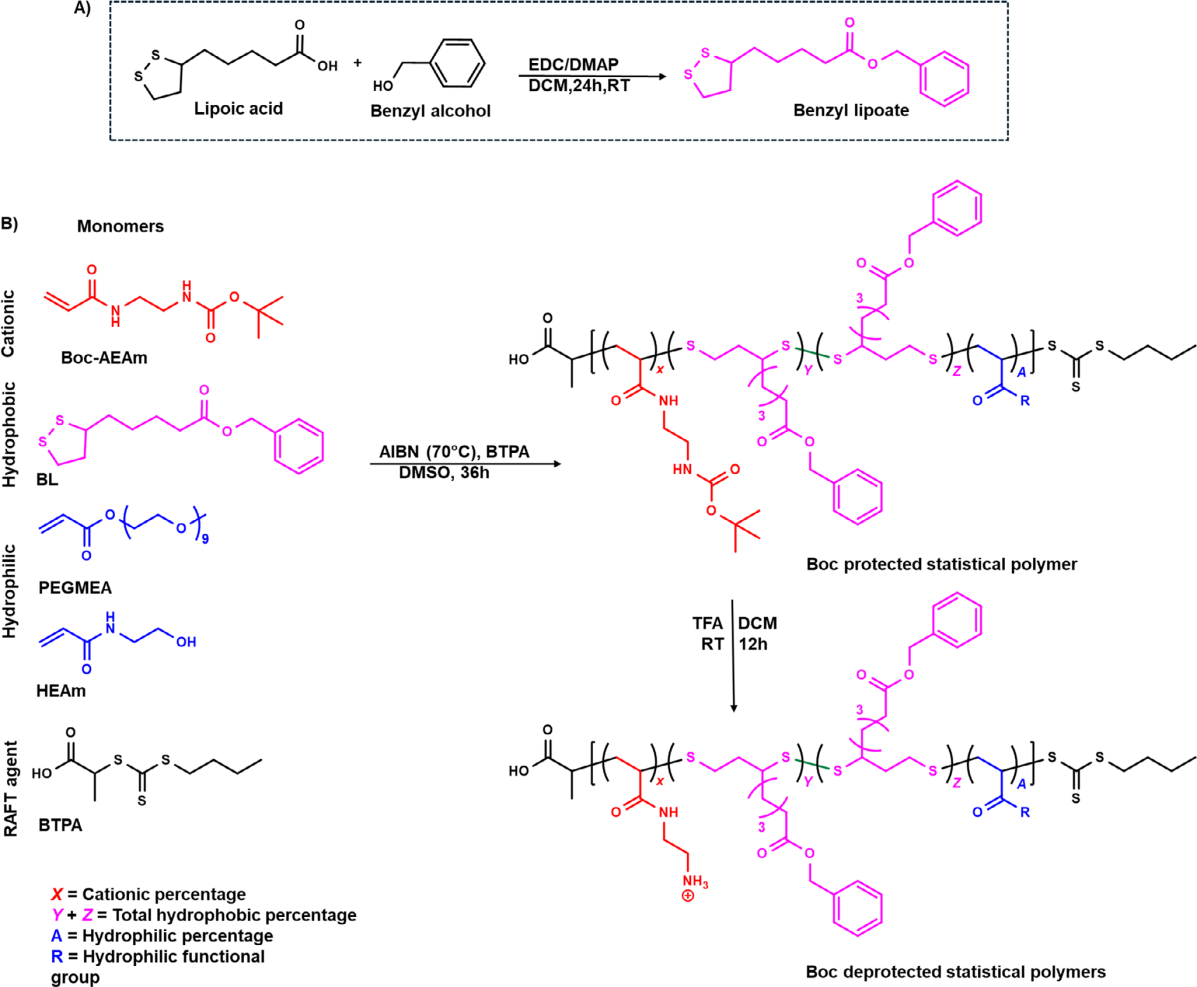
(A) Synthesis scheme of Benzyl lipoate (BL) using EDC/DMAP coupling reaction; (B) schematic illustration of antimicrobial polymer (AP) synthesis using thermal RAFT process, followed by polymer deprotection using trifluoroacetic acid (TFA).

Building upon previous reports on LA copolymerization with vinyl monomers via RAFT polymerization [94, 95, 96], we selected this technique for the synthesis of APs. We prepared a series of BL‐based antimicrobial polymers by copolymerizing BL with *tert*‐butyl (2‐acrylamidoethyl) carbamate (Boc‐AEAm) (Figures S3 and S4) used as masked cationic monomer, with varying amount of either 2‐hydroxyethyl acrylamide (HEAm) (Figures S5 and S6) or poly(ethylene glycol) methyl ether acrylate (PEGMEA) (Figures S7 and S8) as hydrophilic monomers (Figure 1). Thermal RAFT polymerization was carried out using azobisisobutyronitrile (AIBN) as a radical initiator and 2‐(butylthiocarbonothioylthio)propanoic acid (BTPA) (Figures S9 and S10) as chain transfer agent. The reaction proceeded at 70 °C for 36 h, targeting a DP of 50. Two distinct families of APs, differing in their hydrophilic components, were synthesized: the HEAm‐family (Figures S11–S14), denoted as Am polymers, and the PEGMEA‐family (Figures S15–S18), based on PEGMEA designated as Ac polymers. A nomenclature system of Am1040 was adopted, where Am indicates to the polymer family (Am or Ac), while 10 and 40 represent the molar percentages of the hydrophilic and hydrophobic components, respectively.

For HEAm family, the cationic content was fixed at 50 mol%, while the molar ratios of HEAm and BL were varied from 10:40 to 40:10 (Table 1). After polymerization, crude reaction mixtures were analyzed by ¹H NMR to determine monomer conversions, allowing for calculation of experimental composition and DP. Acrylamide or acrylate conversions were monitored by the decrease of signals between 5.5 and 6.5 ppm attributed to acrylic or acrylamide bonds, and BL was confirmed by the signal shift of benzylic protons from 5.10 to 5.05 ppm. While complete monomer conversions were not achieved after 36 h, the experimental polymer compositions closely matched the initial feed ratios. For instance, in polymer Am2525, the monomer conversions were 78% for the cationic monomer, 66% for HEAm, and 80% for BL (Figure S12), resulting in an experimental composition of 53:21:26 in agreement with the feed ratio of 50:25:25 (see Supporting Information for calculations). In the purified polymers, the presence of the benzylic peak at 5.1 ppm from BL, along with other characteristic comonomer signals associated to hydroxyl group of HEAm at 4.8 ppm and Boc‐deprotected amine (‐NH‐) groups at 8–8.5 ppm, were observed (Figures S13 and S14), confirming the copolymer structures.

**TABLE 1:** Characterization of synthesized antimicrobial polymers using benzyl lipoate (BL) as hydrophobic monomer.

Polymers		Feed ratio Cat.: Hydrophilic: BL	Comp. Cat.: Hydrophilic: BL^a^	DP ^a^	Targeted *M_n_* ^b^ (kg/mol)	*M_n, NMR_* ^c^ (kg/ mol)	*M_n, SEC_* ^d^ (kg/ mol)	*Đ* ^d^	*D*_h_ (nm)^e‐MHB^	*ζ* (mV)^f‐MHB^	*D*_h_ (nm)^e‐PBS^	*ζ* (mV)^f‐PBS^	ζ (mV)^f‐DI‐water^	Abs ^g^
HEAm family	Am1040	50:10:40	52:8:40	20	12.0	5.3	8.6	1.28	540	−4	n.d.	+23	+26	1.8
	Am2030	50:20:30	49:15:36	22	11.2	5.2	9.0	1.30	492	−3	5	+16	+25	1.7
	Am2525	50:25:25	53:21:26	32	10.7	6.5	10.7	1.24	442	−2	5	+6	+29	1.6
	Am3020	50:30:20	53:26:21	38	10.2	7.9	11.8	1.22	400	−2	5	+9	+26	1.5
	Am4010	50:40:10	52:39:9	44	9.3	8.3	13.2	1.17	254	−2	5	+2	+22	0.5
PEGMEA family	Ac1040	50:10:40	47:14:39	25	13.9	7.2	9.6	1.32	197	−5	5	+12	+68	0.2
	Ac2030	50:20:30	45:25:30	31	14.8	9.6	11.7	1.34	21	−3	4	+14	+60	0.1
	Ac2525	50:25:25	48:30:22	38	15.2	12.3	11.2	1.30	25	−3	3	+2	+47	0.1
	Ac3020	50:30:20	42:29:29	51	15.7	16.0	14.6	1.18	34	−4	4	+11	+44	0.1
	Ac4010	50:40:10	44:46:10	44	16.6	15.4	16.0	1.24	28	−6	4	+6	+42	0.1
	Ac3535	30:35:35	25:44:31	29	17.0	10.4	10.7	1.28	44	−8	4	+2	+33	0.1
	Ac3030	40:30:30	37:38:25	36	16.1	12.2	11.7	1.33	24	−4	4	+8	+56	0.1
	Ac2020	60:20:20	58:23:19	43	14.4	12.8	12.8	1.20	19	−3	3	+2	+29	0.1
	Ac1515	70:15:15	71:11:18	43	13.5	11.2	13.3	1.22	50	−5	n.d.	+2	+37	0.1

^a^ Comp. Cat.: Hydrophilic: BL describes the polymer's molar composition in terms of its cationic, hydrophilic, and hydrophobic monomer ratios. This ratio was calculated from ¹H NMR data obtained after 36 h of polymerization (see Supporting Information);

^b^ Theoretical molecular weights of Boc‐protected polymers calculated assuming full monomer conversion;

^c^ Experimental molecular weights of Boc‐protected polymers calculated by ¹H NMR;

^d^ Determined by size exclusion chromatography of Boc‐protected polymers without purification using DMAc eluent;

^e‐MHB^ DLS Particle size of purified Boc‐deprotected polymers in MHB media (Polymer concentration: 1 mg mL^−1^);

^f‐MHB^ Zeta potential of purified Boc‐deprotected polymers in MHB (Polymer concentration: 1 mg mL^−1^);

^e‐PBS^ DLS Particle size of purified Boc‐deprotected polymers in PBS media (Polymer concentration: 1 mg mL^−1^);

^f‐PBS^ Zeta potential of purified Boc‐deprotected polymers in PBS (Polymer concentration: 1 mg mL^−1^);

**^f‐DI‐Water^ :**Zeta potential of purified Boc‐deprotected polymers in Milli‐Q water (Polymer concentration: 1 mg mL^−1^);

^g^ Abs is turbidity by absorbance measurement at 595 nm in MHB (Polymer concentration: 1 mg mL^−1^); n.d. not determined.

Interestingly, increasing BL content from 10% to 40% in the reaction mixture led to a gradual decrease in monomer conversions for all monomers (Figures S12 and S16). Consequently, the experimental DP consistently decreased with increasing disulfide content. Despite these variations in monomer conversions and DP, the experimental polymer compositions remained in good agreement with the initial feed ratios (Table 1). Similar observations were made for PEGMEA family, where a decrease in monomer conversions and DP (calculated by ^1^H NMR) were observed with an increase in BL content, and a relatively good agreement between feed ratios and compositions was maintained. ^1^H NMR spectra of the purified polymers (for example, Ac polymer) confirm the presence of characteristic signals corresponding to BL at 2.3–3.0 ppm (‐CH_2_‐S) and 5.05 ppm (‐CH_2_‐ benzyl group). Additionally, signals associated with the PEGMEA moiety are observed at 4.2 ppm (‐CH₂‐ adjacent to the ester) and 3.5 ppm (‐CH_2_‐O‐). The presence of Boc‐deprotected amine (‐NH‐) groups is indicated by a signal at 8.0 ppm (Figures S17 and S18, see Supporting Information for further details). This confirmed the successful incorporation of all monomer units into the purified copolymer.

Size exclusion chromatography (SEC) was used to characterize the Boc‐protected polymer using dimethylacetamide (DMAc) as the eluent solvent. The copolymers exhibited relatively low dispersity (*Đ)* values ranging from 1.17 to 1.34, indicating well‐controlled molecular weight distributions and demonstrating the effectiveness of RAFT process (Table 1 and Figure S20). The observed deviations between the experimental number‐average molecular weight determined by SEC (*M_n_*, _SEC_) and by NMR (*M_n_*, _NMR_) were attributed to differences in hydrodynamic volume between the copolymers and the PMMA standard used for SEC calibration (Table 1). Following polymerization, the Boc‐protecting group was directly removed from the unpurified APs using trifluoroacetic acid (TFA) at room temperature for 12 h. The successful deprotection was confirmed via ¹H NMR spectroscopy, as evidenced by the absence of the characteristic methyl proton (Boc group) at 1.42 ppm and urethane group at 8.0 ppm (Figures S13 and S17). The polymer was subsequently purified via multiple precipitations in cold diethyl ether. ¹H NMR analysis of the purified products confirmed their chemical structures and purity (Figure S13 as an example).

These purified, water‐soluble APs were characterized by turbidity measurement (Table 1), dynamic light scattering (DLS), and zeta potential in both water and bacterial growth media. In water, all synthesized polymers were completely soluble and did not form visible aggregates, as confirmed by turbidity measurements, indicating their excellent colloidal stability. Zeta potential (ζ) analysis showed that all Boc‐deprotected polymers possessed a positive surface charge (+2 to +23 mV) in PBS, consistent with the presence of primary ammonium ions in their structures. In contrast, in Mueller–Hinton Broth (MHB), the medium used for bacterial growth, the polymers exhibited a negative surface charge (−2 to −8 mV). This observed negative charge in MHB (ζ‐_MHB_ is −16 mV) is likely due to the adsorption of negatively charged components from the complex broth medium onto the intrinsically positive polymer surface. However, upon contact with negatively charged bacterial membranes, these adsorbed negative components can be displaced, allowing the polymers to interact with the bacterial membranes more effectively [16]. Particle size analysis using DLS showed that the HEAm polymer family exhibited larger hydrodynamic diameters (*D*_h_), ranging from 254 to 540 nm compared to the PEGMEA family in MHB, which typically ranged from 20 to 50 nm, with the exception of Ac1040 (Table 1). Furthermore, turbidity measurements indicated that the PEGMEA polymer family exhibited superior solubility in MHB (Table 1). Due to the presence of cationic primary amine and hydrophobic groups, these polymers can interact with proteins, leading to aggregation. However, the presence of neutral hydrophilic groups help mitigate these non‐specific interactions and reduce aggregation [41, 97]. Despite the presence of HEAm, our results demonstrated that polymers from the HEAm family formed protein‐polymer complexes in MHB, as evidenced by the presence of large aggregates (Figure S21). In contrast, PEGMEA‐based polymers remained well‐dispersed, likely due to the longer PEG chains effectively shielding hydrophobic groups and reducing unwanted non‐specific protein interactions.

### 2.2 Antibacterial Activity

The antibacterial activity of the purified synthesized APs was assessed by determining the minimum inhibitory concentration (MIC_90_), defined as the lowest concentration required to inhibit 90% of bacterial growth relative to untreated controls. MIC assays were conducted against four Gram‐negative bacterial strains, including *Escherichia coli* K12, *Pseudomonas aeruginosa* ATCC 27853 and PA37, and *Acinetobacter baumannii* ATCC 19606 as well as one Gram‐positive bacterial strain, *Staphylococcus aureus* ATCC 29213.

### 2.3 HEAm‐Based Polymers

Within the HEAm‐polymer family, Am1040 (40% BL) exhibited a MIC of 128 µg mL^−1^ against *E. coli*. A 10% reduction in the BL% from Am1040 to Am2030 increased in antibacterial activity against *E. coli*, lowering MIC to 64 µg/mL (Table 2). However, a further reduction in BL content, for example Am2525 (25% BL) and Am4010 (10% BL) led to a significant reduction in antibacterial efficacy against *E. coli*, with the MIC increasing from 128 to >256 µg mL^−1^. Unlike the trend observed for *E. coli*, all HEAm‐based polymers exhibited an MIC of 128 µg mL^−1^ against *P. aeruginosa* ATCC 27853, while no antibacterial activity was detected against *P. aeruginosa* PA37 and *A. baumannii* at the highest tested concentration. Moreover, the representative polymer Am2525 exhibited no detectable antibacterial activity against the Gram‐positive strain *Staphylococcus aureus* (SA, ATCC 29213), even at the highest concentration tested (Table S2). This relatively low activity was attributed to the complexation of APs with proteins in MHB as suggested by turbidity and DLS measurements.

**TABLE 2:** Antibacterial activity (MIC_90_) and hemolysis (HC_20_) values for the synthesized polymers.

Polymers		Feed ratio Cat.: Hydrophilic: BL	MIC_90_				HC_20,2 h_ (µg/mL)	SI PA‐ATCC [HC_20_ / MIC_90_]
			[EC] (µg/mL)	[PA‐ATCC] (µg/mL)	[PA37] (µg/mL)	[AB] (µg/mL)		
HEAm family	Am1040	50:10:40	128	128	>256	>256	<125	<1
	Am2030	50:20:30	64	128	>256	>256	<125	<1
	Am2525	50:25:25	128	128	>256	>256	<125	<1
	Am3020	50:30:20	128	128	>256	>256	<125	<1
	Am4010	50:40:10	>256	256	>256	>256	>2000	>7
PEGMEA family	Ac1040	50:10:40	64	32–64	64	64	<125	<1
	Ac2030	50:20:30	32–64	32	128	32	<125	<1
	Ac2525	50:25:25	64	32	128	32‐64	>2000	>62
	Ac3020	50:30:20	64–128	64 – 128	128‐256	128	>2000	>15
	Ac4010	50:40:10	>256	256	>256	256	>2000	>7
	Ac3535	30:35:35	256	>256	>256	128	>2000	<1
	Ac3030	40:30:30	64	64	128–256	64	>2000	>31
	Ac2020	60:20:20	64–128	64	64	>256	>2000	>31
	Ac1515	70:15:15	64–128	64–128	128	>256	1000	>7

*Note*: Antibacterial activity (MIC_90_) after 20 h of incubation; Hemolysis activity against RBCs (HC_20_) after 2 h of incubation; Selectivity index (SI_PA_) is the ratio of HC_20_ against MIC_90_ (PA‐ATCC); EC – *E. coli* K12; PA‐ATCC – *P. aeruginosa* ATCC 27853; PA37 – *P. aeruginosa* P37; AB – *A. baumannii* ATCC 19606).

### 2.4 PEGMEA‐Based Polymers

For PEGMEA‐based polymers, Ac1040 (40% BL) exhibited an MIC of 64 µg mL^−1^ against *E. coli* (Table 2). A 10% reduction in BL content (Ac2030) slightly improved antibacterial activity, with MIC ranging from 32 to 64 µg mL^−1^ against *E. coli*. However, further reducing in BL content to 10% (Ac4010) resulted in complete loss of activity against *E. coli*, following a similar trend to that observed in the HEAm polymer family. For *P. aeruginosa* ATCC 27853, Ac1040 (BL 40 mol%) displayed an MIC of 32–64 µg mL^−1^. A moderate reduction in BL% (Ac2030 to Ac2525) yielded a consistent MIC of 32 µg mL^−1^. However, further decreasing the BL content resulted led to a significant loss of activity, with the MIC increasing to 256 µg mL^−1^. We also evaluated the antibacterial activity of PEGMEA‐based polymers against *P. aeruginosa* PA37 and *A. baumannii*. Ac1040 showed an MIC of 64 µg mL^−1^ for both strains. However, as previously observed, a reduction in BL content corresponded to decreased antimicrobial activity. For example, a 10 mol% reduction in BL content (Ac2030) resulted in an MIC increase to 128 µg/mL for *P. aeruginosa* PA37. When the BL mol% was further reduced to 10% (Ac4010), antibacterial activity was negligible against both strains. Moreover, the selected polymer Ac2525 displayed no observable antibacterial effect against the Gram‐positive strain SA, ATCC 29213, even at the highest tested concentration (Table S2). This disparity in antibacterial efficacy between Gram‐negative and Gram‐positive bacteria can be attributed to fundamental differences in their cell membrane architecture [98, 99]. Gram‐negative bacteria possess an outer membrane enriched with lipopolysaccharides and a relatively thin, loosely cross‐linked peptidoglycan layer, which may facilitate electrostatic interactions and membrane disruption by the cationic and hydrophobic domains of the terpolymers. In contrast, the thick, highly cross‐linked peptidoglycan structure of Gram‐positive bacteria acts as a selective barrier, limiting the diffusion of amphiphilic macromolecules and impeding their access to the cytoplasmic membrane, thereby reducing antimicrobial efficacy [42]. Overall, PEGMEA‐based polymers demonstrated broader antimicrobial activity across different Gram‐negative bacteria compared to HEAm‐based polymers, which exhibited only modest activity against *E. coli*.

### 2.5 Hemocompatibility

To evaluate the hemocompatibility of BL‐based APs, we conducted a hemolysis assay using sheep red blood cells (RBCs) and determined HC_20_ values, defined as the lowest polymer concentration causing no more than 20% hemolysis. Within the HEAm polymer family, Am1040 (containing 40 mol% BL) exhibited RBC toxicity, with an HC_20_ value of <125 µg mL^−1^ (Table 2). Reducing the BL content from Am1040 (40%) to Am3020 (20%) did not significantly change HC_20_, as the HC_20_ value remained below 125 µg mL^−1^. This suggests that incorporating up to 30 mol% of HEAm was insufficient to achieve an optimal amphiphilic balance to mitigate hemolysis. Notably, only Am4010, which contains 10% BL, exhibited hemocompatibility, with an HC_20_ value exceeding 2000 µg mL^−1^ (the highest concentration tested).

Subsequently, we tested PEGMEA family. Similar to the HEAm family, PEGMEA‐based polymers with 40% (Ac1040) and 30% (Ac2030) BL exhibited high hemolytic activity, with HC_20_ values of <125 µg mL^−1^ (Table 2). However, a slight reduction in BL content from Ac2030 (30% BL) to Ac2525 (25% BL) improved hemocompatibility, with an HC_20_ value >2000 µg mL^−1^. Furthermore, all PEGMEA‐based polymers containing 25% or less of BL displayed HC_20_ values above 2000 µg mL^−1^, highlighting their superior hemocompatibility compared to the HEAm polymer family. These results emphasize the critical role of hydrophilic groups in reducing RBC hemolysis and align with previous studies demonstrating that increasing APs hydrophobicity enhances RBC membrane disruption [16, 68].

### 2.6 Optimization of Antibacterial Activity and Hemocompatibility

A comparison between the two polymer families revealed that Ac2525 demonstrated superior hemocompatibility and antimicrobial activity compared to Am2525 at an equivalent hydrophilic‐to‐hydrophobic ratio (25% BL). This enhanced performance is due to the difference in structural components. Specifically addressing the roles of the PEG chain and the benzyl group in the overall selective antibacterial action of Ac2525: The PEG chain primarily enhances the polymer's hydrophilicity and aqueous solubility. While this significantly improves hemocompatibility by reducing nonspecific interactions with mammalian cell membranes (like RBCs), it also plays a crucial role in the antibacterial mechanism by ensuring the polymer remains soluble and available to interact effectively with bacteria in an aqueous environment, contributing to overall bioavailability and selectivity. In contrast, the benzyl group provides the essential hydrophobic character. This hydrophobicity is the primary driver for promoting strong interactions with the lipid components of bacterial membranes, leading to membrane disruption and potent antibacterial killing. Thus, the PEG chain facilitates selectivity and bioavailability, while the benzyl group directly mediates the antibacterial mechanism by disrupting the membrane. The observed differences underscore the importance of achieving a balanced between cationic, hydrophilic (PEG), and hydrophobic (BL) components. Such balance is crucial for optimizing selective antibacterial activity while minimizing hemotoxicity toward red blood cells. Additionally, the selectivity index (SI_PA_) calculated using the MIC against *P. aeruginosa* ATCC 27853 and HC_20_ identified Ac2525 as the most potent polymer (SI_PA_ >62), effectively balancing antibacterial efficacy and RBC compatibility (Table 2). Interestingly, BL‐based polymers demonstrated improved hemocompatibility compared to previously reported linear polymers with similar compositions, but containing ethyl hexyl acrylate or benzyl acrylamide as hydrophobic groups [46]. Furthermore, Ac2525 exhibited no detectable hemagglutination based on visual assessment, (Table S3) which indicated that a PEGMEA content of 25% or higher effectively prevented hemagglutination, highlighting its crucial role in enhancing hemocompatibility.

To investigate the impact of cationic content on the hemocompatibility and antibacterial activity of BL‐based polymers, we selected Ac2525 as a model polymer due to its high selectivity index (SI_PA_). By maintaining an equimolar ratio of hydrophobic BL and PEGMEA, we systematically varied the cationic content from 30 mol% to 70 mol%. The antimicrobial efficacy of these polymers (Ac3535, Ac3030, Ac2020 and Ac1515), each with an equal hydrophilic‐to‐hydrophobic ratio, was evaluated against our four bacterial strains. Polymer Ac3535 (cationic: hydrophilic: hydrophobic = 30:35:35) exhibited negligible antibacterial activity, with an MIC of 256 µg mL^−1^ against *E. coli* and >256 µg mL^−1^ against *P. aeruginosa* ATCC 27853. Increasing the cationic content to 40% (i.e., Ac3030) significantly improved antibacterial activity, decreasing the MIC values to 64 µg mL^−1^ for *E. coli* and *P. aeruginosa* ATCC 27853. Further increasing the cationic content to 60% in Ac2020 and 70% in Ac1515 maintained MIC values in the range of 64–128 µg mL^−1^ for *E. coli*, *P. aeruginosa* ATCC 27853 and *P. aeruginosa* PA37. However, Ac2020 and Ac1515 remained inactive against *A. baumannii* at the highest tested concentration. Interestingly, these four polymers displayed good hemocompatibility, with HC_20_ values exceeding 2000 µg mL^−1^ for Ac3535, Ac3030, Ac2020 and 1000 µg mL^−1^ for Ac1515. Additionally, selected polymers from the HEAm (Am2525) and PEGMEA (Ac2525, Ac3030, Ac2020 and Ac1515) families were assessed against the Gram‐positive bacterial strain SA (ATCC 29213); no antibacterial activity was observed, even at the highest concentration tested (Table S2).

Polymers containing 40% and 60% cationic content (Ac3030 and Ac2020) exhibited comparable antimicrobial efficacy, with MIC values ranging from 64 to 128 µg mL^−1^ against Gram‐negative bacterial strains. Incorporation of hydrophobic benzyl lipoate in an equimolar ratio with the hydrophilic PEG segment was critical for promoting membrane disruption and enhancing bactericidal performance. Notably, Ac2525, comprising 50% cationic units, demonstrated superior antibacterial activity and hemocompatibility compared to its analogue Am2525 at an equivalent hydrophilic‐to‐hydrophobic ratio (25% BL). Further increasing the cationic content beyond 60% did not yield additional antimicrobial benefits and was associated with a moderate decline in hemocompatibility. These findings highlight the critical role of optimizing the cationic‐to‐hydrophobic ratio to maximize antimicrobial efficacy while preserving high hemocompatibility (HC_20_ ≥ 1000 µg mL^−1^).

### 2.7 3T3 Fibroblast Cell Toxicity Test

Selected antimicrobial polymers (APs) from the HEAm family (Am2030, Am2525) and the PEGMEA family (Ac1040, Ac2030, Ac2525, Ac3020, Ac3030) were evaluated for cytocompatibility using 3T3 fibroblast cells. Cytotoxicity was assessed by determining the inhibitory concentration (IC_50_), which represents the polymer concentration required to reduce cell viability by 50% after 24 h of incubation, as measured via the MTT assay (see Supporting Information). Among the tested polymers Am2030 (30% BL), Am2525 (25%BL), Ac1040 (40% BL) and Ac2030 (30% BL) terpolymers exhibited the highest cytotoxicity, with IC_50_ values of 100 µg mL^−1^. In contrast, all other terpolymers demonstrated moderate cytotoxicity, with IC_50_ values exceeding 100 µg mL^−1^ (Table S2). For the PEGMEA family, Ac2525 (25% BL) terpolymer displayed moderate cytotoxicity and Ac3020 (20% BL) showed improved cytocompatibility, with IC_50_ values of 200 and 400 µg mL^−1^, respectively. These results highlight the inverse correlation between hydrophobicity and cytotoxicity, suggesting that reduced hydrophobic content improves polymer biocompatibility. A comparative analysis between two amphiphilic balanced polymers, Ac2525 (50% cationic) and Ac3030 (40% cationic), revealed IC_50_ values of 200 and 400 µg mL^−1^, respectively. Although both polymers exhibit amphiphilic balance, a 10% reduction in cationic content resulted in a two‐fold improvement in mammalian cell viability.

To further assess the therapeutic potential of these polymers, we calculated the Selectivity Index for cytotoxicity (SI_C_), defined as the ratio of IC_50_ to MIC against *P. aeruginosa* (see Table S2). The Ac2030 polymer demonstrated an SI_C_ of >3, while the higher IC_50_ of Ac2525 resulted in an enhanced SI_C_ of >6, indicating improved selectivity toward bacterial cells over mammalian cells. However, further reduction in hydrophobic content in Ac3020 led to a diminished antimicrobial efficacy, reflected by a significant increase in MIC values and a subsequent decrease in SI_C_ to >3. When comparing Ac2525 and Ac3030, both of which maintain amphiphilic balance, Ac2525 exhibited superior antibacterial activity with moderate cytotoxicity, whereas Ac3030 showed reduced antibacterial potency but significantly improved cytocompatibility. Based on the SI_C_ values, both polymers demonstrate comparable selectivity index (SI_C_ > 6), showing a balance between antimicrobial activity and biocompatibility. These results highlight the need to carefully adjust amphiphilic balance and charge distribution to develop polymers with optimal therapeutic performance.

### 2.8 Degradation of Antimicrobial Polymers

**FIGURE 2:**
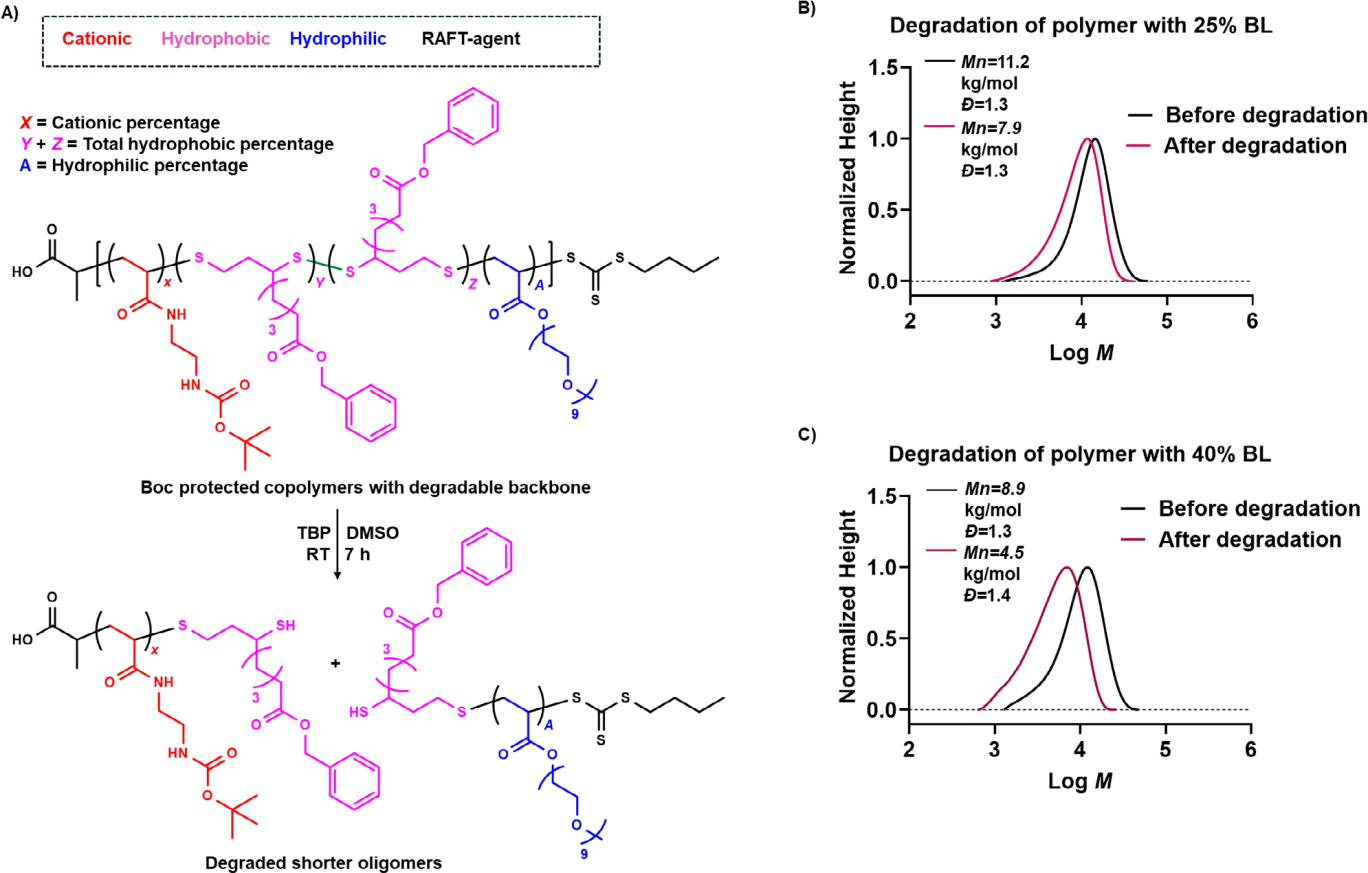
(A) Schematic presentation of polymer degradation; Degradation monitoring by SEC track of (B) 25% BL and (C) 40% BL containing Boc protected polymer after 7 h of incubation with 5 equivalents of tri‐*n*‐butylphosphine (TBP) at room temperature.

Incorporating BL (LA derivate) into the antimicrobial polymers (APs) structure introduces disulfide bonds [80, 94, 100, 101], which can be efficiently cleaved under reducing conditions, facilitating polymer degradation and elimination. To investigate the influence of disulfide bond density on the degradation of our antimicrobial polymers, we synthesized two polymers with varying BL content: 40 mol% BL (Boc‐AEAm: PEGMEA: BL = 40:20:40) and 25 mol% BL (Boc‐AEAm: PEGMEA: BL = 50:25:25). The polymer degradation study was performed using Boc‐protected polymers to enable their SEC characterization. Indeed, after deprotection, these polymers were only soluble in water, but due to the presence of cationic and hydrophobic groups, their analysis by water SEC was not possible. To circumvent these limitations, all degradation characterizations were conducted in an organic solvent (DMAc) using the disulfide‐reducing agent tri‐*n*‐butylphosphine (TBP) (Figure 2). SEC analysis revealed that the polymer containing 40 mol% BL exhibited a more pronounced molecular weight reduction (from 8900 to 4500 g mol^−1^) compared to the 25 mol% BL polymers (from 11 200 to 7900 g mol^−1^) (Figure 2B,C), highlighting the influence of disulfide bond density on degradation behaviour. This trend is further supported by ¹H NMR analysis (Figure S19). The purified Boc‐protected polymer containing 40% BL shows characteristic broad signals at 2.4–2.7 ppm, corresponding to the protons in adjacent position of thioether (─CH_2_─S─) in the polymer backbone. Upon treatment with TBP, the appearance of new peaks at 2.75 ppm, attributed to CH_2_ protons adjacent to SH group confirms the degradation [100, 102] of disulfide bonds (Figure S19). The degraded polymers were subsequently characterized by dynamic light scattering (DLS) to assess its hydrodynamic volume and zeta potential (Table S1).

## 3 Conclusion

A library of 14 copolymers for antibacterial activity and hemocompatibility evaluation and two polymers for degradation studies was successfully synthesized using thermal RAFT polymerization. We investigated the hemocompatibility of two hydrophilic monomers, hydroxyethyl acrylamide (HEAm) and poly(ethylene glycol) methyl ether acrylate (PEGMEA), with benzyl lipoate. Our findings indicate that PEGMEA exhibits superior compatibility, resulting in enhanced antimicrobial activity and improved hemocompatibility. During polymerization involving benzyl lipoate, monomer conversion decreased with increasing benzyl lipoate content. The synthesized polymers demonstrated antimicrobial activity against Gram‐negative bacterial strains but exhibited no activity against Gram‐positive bacteria, even at the highest tested concentration. Among the PEGMEA‐based polymers, Ac2525 demonstrated the most promising antimicrobial activity while maintaining high hemocompatibility. Maintaining a hydrophilic/hydrophobic ratio of 1:1 was found to be crucial for antimicrobial efficacy, as reducing the cationic content below 50 mol% diminished activity, while increasing it to 70 mol% induced hemotoxicity. Additionally, the presence of disulfide bonds within the APs backbone enabled polymer degradation under reducing conditions, highlighting their potential for controlled degradation. This feature has significant implications for minimizing long‐term toxicity in biological systems and improving environmental safety. Lipoic acid‐based degradable APs hold significant potential for antimicrobial applications. Future studies should focus on optimizing their degradation kinetics and antimicrobial performance to develop safer and more effective antimicrobial materials.

## Conflicts of Interest

The authors declare no conflict of interest.

## Supporting information




**Supporting File 1**: marc202500224‐sup‐0001‐SuppMat.docx.

## Data Availability

The data that support the findings of this study are available in the supplementary material of this article.
