# Supplementary material for: Lipoic Acid Based Redox‐Responsive Degradable Antimicrobial Polymers
Source: Macromol Rapid Commun. 2025 Jun 17;46(17):e00224. doi: 10.1002/marc.202500224 (PMC12412199; doi:10.1002/marc.202500224)
Supplement: Supplementary file 1 — Supporting File 1: marc202500224‐sup‐0001‐SuppMat.docx. [file MARC-46-e00224-s001.docx]

**Supporting Information**

**Lipoic acid Based Redox-Responsive Degradable Antimicrobial Polymers**

Anupama Giri,^1,2^ Md Aquib,^2^ Anmol Choudhury,^1,2^ Vinod Kumar Kannaujiya,^1^ Jie Lay Lim,^1^ Zi Gu,^1^ Megan D. Lenardon,^*2^ Cyrille Boyer^*1^

^1^ Cluster for Advanced Macromolecular Design (CAMD) and School of Chemical Engineering, UNSW, Sydney, NSW, 2052, Australia

^2^ School of Biotechnology and Biomolecular Sciences, UNSW, Sydney, NSW, 2052, Australia

E-mails: [cboyer@unsw.edu.au](mailto:cboyer@unsw.edu.au); [m.lenardon@unsw.edu.au](mailto:m.lenardon@unsw.edu.au)

Contents

[Materials and Methods 3](#_Toc198725955)

[**Materials** 3](#_Toc198725956)

[**Synthesis of hydrophobic monomer benzyl lipoate** 3](#_Toc198725957)

[**Synthesis of polymer library** 3](#_Toc198725958)

[**Deprotection of polymers** 4](#_Toc198725959)

[**Degradation study** 4](#_Toc198725960)

[Characterizations 4](#_Toc198725961)

[**NMR spectroscopy** 4](#_Toc198725962)

[**Size-Exclusion Chromatography** 5](#_Toc198725963)

[**Characterization of terpolymers in aqueous media** 5](#_Toc198725964)

[Hemolysis study 6](#_Toc198725965)

[Cytotoxicity test 6](#_Toc198725966)

[Supporting Information – Additional Data 7](#_Toc198725967)

# **Materials and Methods**

## **Materials**

Azobisisobutyronitrile (AIBN) (12 wt. % in acetone, Sigma-Aldrich), benzyl alcohol (Sigma-Aldrich,99%), DL-alpha-Lipoic Acid (Ambeed, 99%), 1-ethyl-3-(3-dimethylaminopropyl)carbodiimide hydrochloride (EDC.HCl, Ambeed, 99 %), 4-(dimethylamino)pyridine (DMAP, Sigma-Aldrich, 99 %), *tert*-butyl (2- acrylamidoethyl)carbamate (Boc-AEAm, Ambeed, 95%), trifluoroacetic acid (TFA; Sigma-Aldrich, 99 %), 2-(butylthiocarbonothioylthio)propanoic acid (BTPA, Boron Molecular, 99%), Colistin sodium methanesulfonate (Sigma-Aldrich), 3-(4,5-dimethyl-2-thiazolyl)-2,5-diphenyl-2H-tetrazolium bromide (MTT, Sigma- Aldrich), Dulbecco’s modified Eagle Medium (DMEM, Sigma-Aldrich), Oxoid™ phosphate buffered saline (PBS) tablet (Thermo Scientific™), *N-*hydroxyethyl acrylamide (HEAm, Sigma Aldrich, 97%), poly(ethylene glycol) methyl ether acrylate (PEGMEA, Sigma-Aldrich, average *M*_n_ = 480 g/mol), deuterated dimethyl sulfoxide (DMSO) (Cambridge Isotope Laboratories, Inc), deuterated acetone (Cambridge Isotope Laboratories, Inc), dichloromethane (DCM; Merck), high purity liquid chromatography (HPLC) grade dimethylacetamide (DMAc, Sigma-Aldrich), were used as received. Acetone, magnesium sulphate (MgSO_4_), diethyl ether, and DMSO, were obtained from Chem-Supply and used as received. Deionized (DI) water was acquired by a Milli-Q water purification system with a resistivity of 18.2 mΩ/cm. Defibrinated sheep red blood cells (RBCs) were purchased from Serum Australis (Australia).

## **Synthesis of hydrophobic monomer benzyl lipoate**

Benzyl Lipoate is the ester of DL-alpha-lipoic acid and benzyl alcohol. It was synthesized by using common 1-ethyl-3-(3-dimethylaminopropyl)carbodiimide (EDC)-coupling method. DL-alpha-lipoic acid (1 eq), benzyl alcohol (1.2 eq) and DMAP (0.1 eq) were dissolved separately in DCM. EDC hydrochloride (1.2 eq) dissolved in DCM was transferred at last in the mixture of reaction kept in 0-5°C. At 25 °C this reaction mixture was carried out for 24 hours. From ¹H NMR and TLC full conversion of DL-alpha-lipoic acid was shown. Excess benzyl alcohol, EDC and DMAP were removed by washing consequently with 0.1 M HCl solution (1 × 75 mL), saturated NaHCO_3_ (1 × 75 mL), brine (1 × 75 mL), and Milli-Q water (1 × 75 mL). DCM was evaporated by rotary evaporator and yellow oily product benzyl lipoate was reprecipitate into DI water (3 times). Final product (approximately 60%) benzyl lipoate was obtained after freeze drying.

## **Synthesis of polymer library**

Our statistical copolymers were synthesised using thermal reversible addition–fragmentation chain transfer (RAFT) polymerization technique. The polymerization technique is previously proposed by various researchers.^1, 2^ In short, a concentration of 33% (w/w) in DMSO was used to generate the monomer stock solutions for tert-butyl (2- acrylamidoethyl)carbamate(Boc-AEAm), 2-ethylhexyl acrylate, benzyl lipoate and poly(ethylene glycol) methyl ether acrylate (PEGMEA). The RAFT agent (BTPA) was dissolved in DMSO and added to a 4 mL glass vial in an amount that matched the intended degree of polymerisation (DP) of 50. In order to reach the desired ratios and a targeted monomer concentration of 25% (w/w) in DMSO, monomer stock solutions were then added to the glass vial. For time zero (T_0_), ¹H NMR sample an aliquot was taken to analyse the initial monomer concentration in polymerization mixture. Thermal radical initiator (AIBN) in acetone was added 0.2 eq to the RAFT agent. A rubber septum was used to securely seal the glass vial, and N_2_ was used to degas the headspace for 20 minutes. The polymerisation was carried out in 70 °C for 36 hours to check the best monomer conversion with benzyl lipoate concentration. Polymerisation was analysed by proton nuclear magnetic resonance (¹H NMR) spectroscopy and size-exclusion chromatography (SEC) to evaluate the monomer conversion, polymer composition, and molecular weight distribution. The crude polymers were deprotected without purification to reduce the loss of polymers in purification steps.

## **Deprotection of polymers**

Deprotection was done by our groups previously proposed method.^3^ Crude polymers was dissolved in DCM (~7% (w/w) polymer) followed by TFA (20 mole equivalent to Boc group). Reaction mixture was stirred for 12 hours at room temperature until complete removal of Boc group as confirmed by ¹H NMR spectroscopy. Boc removed polymers was precipitated (3 times) in ice-cold diethyl ether followed by centrifugation (9000 rpm for 4 mins) and dissolved in acetone and reprecipitated. ¹H NMR analysis was done for the vacuum dry Boc deprotected polymers after removal of most of the solvents.

## **Degradation study**

Tri-*n* butyl phosphine (5 equivalent to disulphide bonds in polymer) was added in crude polymer mixture dissolved in DMSO. The reaction mixture was incubated at room temperature for 7 hours. We used Boc protected crude polymers for degradation study without any purification. Boc-protected polymers and degraded polymers were analyses by size exclusion chromatography (SEC), DMAc as an eluent. The decrease in number average molecular weight was analyses by SEC. After degradation, we purified the polymers and removed the Boc group using our established deprotection and purification method.^3^

# **Characterizations**

## **NMR spectroscopy**

¹H NMR and ^13^C NMR spectra were obtained using a Bruker Avance III (400 MHz) spectrometer equipped with a sampleXpess probe. Deuterated DMSO, chloroform and acetone were used as the solvent, and samples were prepared at a concentration of 5-10 mg/mL. This technique was used to assess monomer purity, polymer composition, and monomer conversion. Before polymerization, a 20 µL aliquot of the unreacted polymerization mixture was analysed to confirm the monomer composition. Data were processed using MestReNova software.

## **Size-Exclusion Chromatography**

SEC analysis was conducted using a Shimadzu liquid chromatography system with a refractive index detector and three MIX C columns. The system operated at 50 °C with a flow rate of 1 mL/min, using DMAc as the eluent, supplemented with LiBr (0.3 g/L) and 2,6-di-butyl-4-methylphenol (0.5 g/L). Calibration was performed with poly(methyl methacrylate) (PMMA) standards (200–10⁶ g/mol). Polymer solutions (25% w/w in DMSO) were diluted in DMAc (3 mg/ml), filtered (0.45 µm), and injected for analysis.

## **Characterization of terpolymers in aqueous media**

Dynamic light scattering (DLS) and zeta potential measurements were conducted on a Malvern Zetasizer Nano ZS, which uses a He–Ne laser with a wavelength of 633 nm and a scattering angle of 173°. Polymer samples were prepared at a concentration of ~ 1 mg/mL in Milli-Q water and Mueller–Hinton broth (MHB). All polymers were filtered through a 0.45 μm syringe filter before aqueous medium analysis. The HEAm polymer family exhibited potential turbidity in MHB media, whereas no visible turbidity was observed for PEGMEA polymers. To ensure accurate measurement of hydrodynamic diameter (*D*_h)_, the polymers were not filtered before MHB media analysis.

**Minimum inhibitory concentration**

The minimum inhibitory concentration (MIC) values of the synthesized antimicrobial polymers and the control drug were determined using the broth microdilution method, following protocols from previous studies.^4, 5^ The test panel included four Gram-negative bacterial strains: *Escherichia coli* (EC K12), *Acinetobacter baumannii* (ATCC 19606), and *Pseudomonas aeruginosa* (PA), including both ATCC 27853 and the multidrug-resistant strain PA37 as well as one Gram-positive strain, *S. aureus* (SA 29213). To prepare for MIC testing, a bacterial culture was grown overnight from a single colony in 10 mL of Mueller–Hinton broth (MHB) at 37 °C with shaking at 180 rpm. A subculture was then prepared by diluting 100 μL of the overnight culture into 10 mL of fresh MHB and grown to mid-log phase (approximately 2.5 hours). This was subsequently diluted to the appropriate concentration. A 2-fold serial dilution of the polymer and colistin samples in MHB was prepared, with 100 μL of each added to wells in a 96-well microplate, followed by 100 μL of the bacterial suspension. The final bacterial concentration in each well was ~5 × 10^5^ cells/mL. Positive controls (with bacteria but no polymer) and negative controls (no bacteria or polymer only MHB) were included in each assay. The plates were incubated at 37 °C for 20 hours, and absorbance was measured at 595 nm using a FLUOstar Omega microplate reader (BMG Labtech). MIC_90_ was defined as the lowest concentration that inhibited 90% of bacterial growth compared to the untreated control. All experiments were performed in independently repeated three times.

# **Hemolysis study**

The hemolytic activity of the polymers and Colistin was assessed using fresh sheep red blood cells (RBCs) based on a method previously established.^6-8^ In brief, RBCs were diluted to a 1:20 ratio in PBS (pH 7.4), pelleted by centrifugation at 4000 g for 10 minutes, and washed three times with PBS. The RBCs were then resuspended to a final concentration of 5% (v/v) in PBS. Various polymer concentrations (150 μL) were prepared in sterilized tubes, and 150 μL of the RBC suspension was added. The polymers and Colistin were tested at concentrations of 2000, 1000, 500, 250, and 125 μg/mL. PBS buffer served as the negative control, while Triton-X 100 (1% v/v in PBS) was used as the positive hemolysis control. The tubes were incubated at 37 °C with shaking at 150 rpm for 2 hours. After incubation, samples were centrifuged at 1000g for 8 minutes, and 100 μL of the supernatant was transferred to a 96-well plate for absorbance measurement at 485 nm using a FLUOstar Omega microplate reader (BMG Labtech). All experiments were conducted in triplicate. The percentage of hemolysis was calculated using the formula:

$$\% Hemolysis =\frac{A_{Polymer} - A_{\mathrm{negative}}}{A_{Positive} - A_{\mathrm{negative}}}\times100 (1)$$

# **Cytotoxicity test**

The cytotoxicity of the selected antimicrobial terpolymers was evaluated using the MTT assay, following established protocols.^9, 10^ Briefly, 3T3 fibroblast cells were cultured in Dulbecco’s Modified Eagle Medium (DMEM) and seeded into flat-bottom 96-well plates at a density of 5 × 10⁴ cells/mL in 100 µL of medium per well. The plates were incubated for 24 hours at 37 °C in a humidified atmosphere containing 5% CO₂ to facilitate cell attachment. Following incubation, the medium was replaced with fresh DMEM supplemented with the polymers at different concentrations (100, 200, 400, 800 µg/mL). Plates were incubated for an additional 24 hours under the same conditions. Control wells received fresh DMEM without polymer to serve as untreated controls. After the exposure period, the medium was aspirated and replaced with 100 µL of MTT solution. The plates were then incubated in the dark at 37 °C for 4 hours, allowing viable cells to reduce MTT into insoluble formazan crystals. Subsequently, the MTT solution was removed, and 100 µL of DMSO was added to each well to solubilize the formazan. The plates were gently shaken for 10 minutes to ensure complete dissolution. The absorbance was measured at 570 nm using a microplate reader, with blank wells included to correct for background signal. Cell viability was calculated as a percentage relative to untreated control wells. IC_50_ defined as the polymer concentration required to reduce cell viability by 50% after 24 hours of exposure. All experiments were performed in triplicate.

# **Supporting Information – Additional Data**

**Figure S1**. ^1^H NMR spectrum of benzyl lipoate (BL, hydrophobic monomer), recroded in DMSO-d_6._ The assigned positions of the monomer's protons are represented by labels **a** to **i**.

**Figure S2**. ^13^C NMR spectrum of benzyl lipoate (BL, hydrophobic monomer), recroded in DMSO-d_6_ (*).

**Figure S3****.** ^1^H NMR spectrum of Boc-AEAm (cationic monomer), recroded in DMSO-d_6._ The assigned positions of the monomer's protons are represented by labels **a** to **g**.

**Figure S4.** ^13^C NMR spectrum of Boc-AEAm (cationic monomer), recroded in DMSO-d_6_ (*).

**Figure S5**. ^1^H NMR spectrum of hydroxyl ethyl acrylamide (HEAm, hydrophilic monomer), recorded in DMSO-d_6._ The assigned positions of the monomer's protons are represented by labels **a** to **f**.

**Figure S6**. ^13^C NMR spectrum of hydroxyl ethyl acrylamide (HEAm, hydrophilic monomer), recroded in DMSO-d_6_ (*).

**Figure S7**. ^1^H NMR spectrum of polyethylene glycol methyl ether acrylate (PEGMEA, hydrophilic monomer), recorded in DMSO-d_6._ The assigned positions of the monomer's protons are represented by labels **a** to **g**.

**Figure S8**. ^13^C NMR spectrum of polyethylene glycol methyl ether acrylate (PEGMEA, hydrophilic monomer), recroded in DMSO-d_6_ (*).

**Figure S9**. ^1^H NMR spectrum of 2-(butylthiocarbonothioylthio)propanoic acid (BTPA, RAFT-agent), recorded in DMSO-d_6._ The assigned positions of the RAFT-agent's protons are represented by labels **a** to **f**.

**Figure S10**. ^13^C NMR spectrum of 2-(butylthiocarbonothioylthio)propanoic acid (BTPA, RAFT-agent), recroded in DMSO-d_6_ (*).

**Figure S11.** ^1^H NMR spectrum of the reaction mixture of Am2525 at T = 0, recorded in DMSO-d_6_.

% Cationic monomer = $\frac{(\int Peak a-\int Peak c)}{(\int Peak a-\int Peak c)+\int Peak c+\frac{\int\mathrm{Peak}b}{2}}$ =$\frac{(31.19-10.03)}{\left( 31.19-10.03 \right)+10.03+\frac{20.02}{2}}$ ×100% = 51.36%

% HEAm monomer = $\frac{\int Peak c}{(\int Peak a-\int Peak c)+\int Peak c+\frac{\int\mathrm{Peak}b}{2}}$ =$\frac{10.03}{\left( 31.19-10.03 \right)+10.03+\frac{20.02}{2}}$ ×100% = 24.34%

% Benzyl lipoate monomer = $\frac{\frac{\int\mathrm{Peak}b}{2}}{(\int Peak a-\int Peak c)+\int Peak c+\frac{\int\mathrm{Peak}b}{2}}$ =$\frac{10.01}{\left( 31.19-10.03 \right)+10.03+\frac{20.02}{2}}$ ×100% = 24.30%

Theoretical DP calculated using T_0_ = 41.2

***Note:*** The signal at 0.86 ppm, corresponding to the -CH₃ group of BTPA, was used as a reference and normalized to an integral value of 3.

**Figure S12.** ^1^H NMR spectrum of the crude Am2525 polymer reaction mixture after 36 hours polymerization, recorded in DMSO-d_6_.

% Monomer conversion(Cationic) = $100\%-\left( \frac{\int{Peak a}_{Pol -}\int{Peak c}_{Pol}}{\int{Peak a}_{T0} -\int{Peak c}_{T0}} \right)\times100\%$

$=100\%-\left( \frac{7.9-3.4}{31.19-10.03} \right)\times100\%$= 78.73%

% Monomer conversion(HEAm) = $100\%-\left( \frac{\int{Peak c}_{Pol}}{\int{Peak c}_{T0}} \right)\times100\%=100\%-\left( \frac{3.40}{10.03} \right)\times100\%$= 66.10%

% Monomer conversion(Benzyl lipoate) = $100\%-\left( \frac{\int{\frac{{Peak b}_{Pol}}{2}}}{\int{\frac{{Peak b}_{T0}}{2}}} \right)\times100\%=100\%-\left( \frac{1.985}{10.01} \right)\times100\%$= 80.17%

Calculated DP after polymerization = 32.2

Percentage of Incorporation (Cationic) = (%Cationic Mole)*(Cationic Conversion)

= 51.36 * $\left( \frac{78.73}{100} \right)$ = 40.43

Percentage of Incorporation(HEAm) = (% HEAm Mole)*(HEAm Conversion) = 24.34 * $\left( \frac{66.10}{100} \right)$ = 16.08

Percentage of Incorporation(Benzyl lipoate) = (% Benzyl lipoate Mole)*(Benzyl lipoate Conversion)

= 24.3 * $\left( \frac{80.17}{100} \right)$ = 19.48

% Cationic monomer in Crude Polymers = $\frac{\text{Percentage of Incorporation (Cationic)}}{\text{Total Percentage of Incorporation}}$

=$\frac{40.43}{(40.43+16.08+19.48)}$ ×100% = 53%

% HEAm monomer in Crude Polymers = $\frac{\text{Percentage of Incorporation (HEAm)}}{\text{Total Percentage of Incorporation}}$

=$\frac{16.18}{(40.43+16.08+19.48)}$ ×100% = 21.16%

% Benzyl lipoate monomer in Crude Polymers = $\frac{\text{Percentage of Incorporation (Benzyl lipoate)}}{\text{Total Percentage of Incorporation}}$

=$\frac{19.48}{(40.43+16.08+19.48)}$ ×100% = 25.6%

***Note:*** The decrease in the vinyl peak at 5.55 ppm, along with the appearance of broad peaks, indicates successful polymerization.

**Figure S13.** ^1^H NMR spectrum of purified Am2525 polymer after Boc-removal and purification, recorded in DMSO-d_6_.

**Figure S14.** ^1^H NMR spectrum of purified Am2525 polymer after Boc-removal and purification, recorded in acetone-d_6_.

**Figure S15.** Example of ^1^H NMR spectrum of the reaction mixture of Ac2525 polymer, recorded in DMSO-d_6_. Reaction mixture (time = 0 hours calculation).

% Cationic monomer = $\frac{\int Peak b}{\int Peak b+\int Peak a+\frac{\int\mathrm{Peak}c}{2}}$ =$\frac{23.13}{23.13+12.15+\frac{22.58}{2}}$ ×100% = 49.66%

% PEGMEA monomer = $\frac{\int Peak a}{\int Peak b+\int Peak a+\frac{\int\mathrm{Peak}c}{2}}$ =$\frac{12.15}{23.13+12.15+\frac{22.58}{2}}$ ×100% = 26.08%

% Benzyl lipoate monomer = $\frac{\frac{\int\mathrm{Peak}c}{2}}{\int Peak b+\int Peak a+\frac{\int\mathrm{Peak}c}{2}}$ =$\frac{\frac{22.58}{2}}{23.13+12.15+\frac{22.58}{2}}$ ×100% = 24.24%

Theoretical DP calculated using T_0_ = 46.57

***Note:*** The signal at 0.86 ppm, corresponding to the CH₃ group of BTPA, was used as a reference and normalized to an integral value of 3.

**Figure S16**. ^1^H NMR spectrum of the crude reaction mixture of Ac2525 polymer, recorded in DMSO-d_6_. Crude polymer reaction mixture (after 36 hours).

% Monomer conversion(Cationic) = $100\%-\left( \frac{\int{Peak b}_{Pol}}{\int{Peak b}_{T0}} \right)\times100\%=100\%-\left( \frac{4.95}{23.13} \right)\times100\%$= 78.6%

% Monomer conversion(PEGMEA) = $100\%-\left( \frac{\int{Peak a}_{Pol}}{\int{Peak a}_{T0}} \right)\times100\%=100\%-\left( \frac{0.57}{12.15} \right)\times100\%$= 95%

% Monomer conversion(Benzyl lipoate) = $100\%-\left( \frac{\int{\frac{{Peak c}_{Pol}}{2}}}{\int{\frac{{Peak c}_{T0}}{2}}} \right)\times100\%=100\%-\left( \frac{2.86}{11.29} \right)\times100\%$= 74.66%

DP after Polymerization = 38.57

Percentage of Incorporation (Cationic) = (%Cationic Mole)*(Cationic Conversion) = 49.66 * $\left( \frac{78.6}{100} \right)$ = 39

Percentage of Incorporation(PEGMEA) = (% PEGMEA Mole)*(PEGMEA Conversion) = 26.08 * $\left( \frac{95}{100} \right)$ = 24.7

Percentage of Incorporation(Benzyl lipoate) = (% Benzyl lipoate Mole)*(Benzyl lipoate Conversion)

= 24.24 * $\left( \frac{74.66}{100} \right)$ = 18.09

% Cationic monomer in Crude Polymers = $\frac{\text{ Percentage of Incorporation (Cationic)}}{\text{ Total Percentage of Incorporation}}$ =$\frac{39}{(39+24.7+18.09)}$ ×100% = 47.67%

% PEGMEA monomer in Crude Polymers = $\frac{\text{Percentage of Incorporation (PEGMEA)}}{\text{Total Percentage of Incorporation}}$ =$\frac{24.7}{(39+24.7+18.09)}$ ×100% = 30.1%

% Benzyl lipoate monomer in Crude Polymers = $\frac{\text{Percentage of Incorporation (Benzyl lipoate)}}{\text{Total Percentage of Incorporation}}$

=$\frac{18.09}{(39+24.7+18.09)}$ ×100% = 22.1%

***Note:*** The decrease in the vinyl peak at 5.55 ppm, along with the appearance of broad peaks, indicates successful polymerization.

**Figure S17.** ^1^H NMR spectrum of purified Ac2525 polymer after Boc-removal and purification. Recorded in DMSO-d_6_

**Figure S18.** ^1^H NMR spectrum of purified Ac2525 polymer after Boc-removal and purification. Recorded in acetone-d_6_.

**Figure S19.** ^1^H NMR spectra of 40 mol% BL (Boc-AEAm: PEGMEA: BL = 40:20:40) containing Boc-protected polymer before and after degradation by tri-*n*-butylphosphine (TBP). Recorded in CDCl_3_.

***Note:*** The appearance of peaks at 2.75 ppm in the Boc-protected polymer after treatment with TBP corresponds to the assigned (*) protons.

**Figure 20.** Molecular weight distributions of Boc-protected APs A) HEAm family; B) PEGMEA family and C) PEGMEA family with equal molar percentages of hydrophilic and hydrophobic components.

**Solubility of polymers in MHB media**

**Figure S21.** Solubility test A) HEAm family polymers B) PEGMEA family polymers in MHB (1mg/mL).

**Table S1:** Characterization of Boc-deprotected purified polymers before and after degradation by DLS, average hydrodynamic diameter (*D*_h_) in MHB, PBS media and zeta potential (*ζ*) in MHB, PBS and Milli-Q water.

| **Polymers:**  **(mol % of BL)** | **Polymers** | *D*_h_ (nm)^e-MHB^ | *ζ*  (mV)^f-MHB^ | *D*_h_ (nm)^e-PBS^ | *ζ*  (mV)^f-PBS^ | *ζ*  (mV)^f-DI-Water^ |
| --- | --- | --- | --- | --- | --- | --- |
| 25% BL | Before degradation | 25 | -3.0 | 3 | +2 | +47 |
|  | After degradation | 24 | -6.0 | 4 | +2 | +50 |
| 40% BL | Before degradation | 32 | -6.0 | 5 | +16 | +53 |
|  | After degradation | 23 | -6.0 | 4 | +14 | +49 |

**Table S2:** Antibacterial Activity (MIC_90_) for selected APs against Gram-positive bacteria (*S. aureus* SA, 29213), Gram-negative bacteria (*P. aeruginosa* PA, ATCC 27853), cytotoxicity value (IC_50_) and selectivity index based on cytotoxicity (SI_C_).

| **Polymers** | | **Feed ratio Cat.: Hydrophilic: BL** | **MIC_90_** | | **IC_50_**  **(µg/mL)** | **SI_C_**  **(IC_50_/MIC_90_)** |
| --- | --- | --- | --- | --- | --- | --- |
|  |  |  | **(SA)**  **(µg/mL)** | **(PA-ATCC)**  **(µg/mL)** |  |  |
| **HEAm family** | Am1040 | 50:10:40 | n.d. | 128 | n.d. | n.d. |
|  | Am2030 | 50:20:30 | n.d. | 128 | n.d. | n.d. |
|  | Am2525 | 50:25:25 | >256 | 128 | 100 | <1 |
|  | Am3020 | 50:30:20 | n.d. | 128 | 100 | <1 |
|  | Am4010 | 50:40:10 | n.d. | 256 | n.d. | n.d. |
| **PEGMEA family** | Ac1040 | 50:10:40 | n.d. | 32-64 | 100 | >1 |
|  | Ac2030 | 50:20:30 | n.d. | 32 | 100 | >3 |
|  | Ac2525 | 50:25:25 | >256 | 32 | 200 | >6 |
|  | Ac3020 | 50:30:20 | n.d. | 64 - 128 | 400 | >3 |
|  | Ac4010 | 50:40:10 | n.d. | 256 | n.d. | n.d. |
|  | Ac3535 | 30:35:35 | n.d. | >256 | n.d. | n.d. |
|  | Ac3030 | 40:30:30 | >256 | 64 | 400 | >6 |
|  | Ac2020 | 60:20:20 | >256 | 64 | n.d. | n.d. |
|  | Ac1515 | 70:15:15 | >256 | 64-128 | n.d. | n.d. |

***Notes*:** Feed ratio Cat.: Hydrophilic: BL denotes the polymer composition, specifically the ratio of cationic, hydrophilic, and hydrophobic groups. The tested polymers showed no activity against the Gram-positive bacterium *S. aureus* (SA, ATCC 29213) even at the highest tested concentration, consistent with our previous findings.^11, 12^ Selectivity index based on cytotoxicity (SI_C_) is the ratio of IC_50_ against MIC_90_ (PA-ATCC). n.d. is not determined.

**Table S3:** Hemagglutination test of RBCs for different concentrations of polymers after 2 hours incubation at 37 °C.

| **Polymers** | | **Feed ratio Cationic: Hydrophilic: BL** | **Hemagglutination concentrations (μg/mL)** | | | |
| --- | --- | --- | --- | --- | --- | --- |
|  |  |  | **2000 μg/mL** | **1000 μg/mL** | **500 μg/mL** | **250 μg/mL** |
| **HEAm family** | Am1040 | 50:10:40 | + | + | + | + |
|  | Am2030 | 50:20:30 | + | + | + | + |
|  | Am2525 | 50:25:25 | + | + | + | + |
|  | Am3020 | 50:30:20 | + | + | + | + |
|  | Am4010 | 50:40:10 | +/– | – | – | – |
| **PEGMEA family** | Ac1040 | 50:10:40 | + | + | + | + |
|  | Ac2030 | 50:20:30 | + | + | + | + |
|  | Ac2525 | 50:25:25 | – | – | – | – |
|  | Ac3020 | 50:30:20 | – | – | – | – |
|  | Ac4010 | 50:40:10 | – | – | – | – |
|  | Ac3535 | 30:35:35 | – | – | – | – |
|  | Ac3030 | 40:30:30 | – | – | – | – |
|  | Ac2020 | 60:20:20 | +/– | – | – | – |
|  | Ac1515 | 70:15:15 | +/– | +/– | +/– | – |

***Notes*:** Hemagglutination was visually assessed,^13^ followed by a hemolysis test, with the results categorized as: + major hemagglutination due to hemolysis; +/– low to moderate hemagglutination; – no detectable hemagglutination.

**Supporting Information - References**

(1) Xiao, M.; Zhang, L.; Tan, J. Transitioning from Photoinitiation to Thermal Initiation in RAFT Dispersion Polymerization via a Small Modification of the Macro-RAFT Agent: A Scalable Approach for Monodisperse Surface-Functional Polymeric Microspheres. *Macromolecules* **2023**, *56* (19), 7675-7688. DOI: 10.1021/acs.macromol.3c01570.

(2) Albanese, K. R.; Morris, P. T.; Read de Alaniz, J.; Bates, C. M.; Hawker, C. J. Controlled-Radical Polymerization of α-Lipoic Acid: A General Route to Degradable Vinyl Copolymers. *Journal of the American Chemical Society* **2023**, *145* (41), 22728-22734. DOI: 10.1021/jacs.3c08248.

(3) Judzewitsch, P. R.; Zhao, L.; Wong, E. H. H.; Boyer, C. High-Throughput Synthesis of Antimicrobial Copolymers and Rapid Evaluation of Their Bioactivity. *Macromolecules* **2019**, *52* (11), 3975-3986. DOI: 10.1021/acs.macromol.9b00290.

(4) Nguyen, T.-K.; Lam, S. J.; Ho, K. K. K.; Kumar, N.; Qiao, G. G.; Egan, S.; Boyer, C.; Wong, E. H. H. Rational Design of Single-Chain Polymeric Nanoparticles That Kill Planktonic and Biofilm Bacteria. *ACS Infectious Diseases* **2017**, *3* (3), 237-248. DOI: 10.1021/acsinfecdis.6b00203.

(5) Namivandi-Zangeneh, R.; Sadrearhami, Z.; Dutta, D.; Willcox, M.; Wong, E. H. H.; Boyer, C. Synergy between Synthetic Antimicrobial Polymer and Antibiotics: A Promising Platform To Combat Multidrug-Resistant Bacteria. *ACS Infectious Diseases* **2019**, *5* (8), 1357-1365. DOI: 10.1021/acsinfecdis.9b00049.

(6) Laroque, S.; Garcia Maset, R.; Hapeshi, A.; Burgevin, F.; Locock, K. E. S.; Perrier, S. Synthetic Star Nanoengineered Antimicrobial Polymers as Antibiofilm Agents: Bacterial Membrane Disruption and Cell Aggregation. *Biomacromolecules* **2023**, *24* (7), 3073-3085. DOI: 10.1021/acs.biomac.3c00150.

(7) Laroque, S.; Locock, K. E. S.; Perrier, S. Cationic Star Polymers Obtained by the Arm-First Approach─Influence of Arm Number and Positioning of Cationic Units on Antimicrobial Activity. *Biomacromolecules* **2025**, *26* (1), 190-200. DOI: 10.1021/acs.biomac.4c00882.

(8) Judzewitsch, P. R.; Nguyen, T.-K.; Shanmugam, S.; Wong, E. H. H.; Boyer, C. Towards Sequence-Controlled Antimicrobial Polymers: Effect of Polymer Block Order on Antimicrobial Activity. *Angewandte Chemie International Edition* **2018**, *57* (17), 4559-4564. DOI: <https://doi.org/10.1002/anie.201713036>.

(9) Deng, Z.; Zhang, R.; Gong, J.; Zhang, Z.; Zhang, L.; Qiu, Z.; Alam, P.; Zhang, J.; Liu, Y.; Li, Y.; et al. Unveiling the Role of Alkyl Chain in Boosting Antibacterial Selectivity and Cell Biocompatibility. *JACS Au* **2025**, *5* (2), 675-683. DOI: 10.1021/jacsau.4c00915.

(10) Li, B.; Hao, G.; Sun, B.; Gu, Z.; Xu, Z. P. Engineering a Therapy-Induced “Immunogenic Cancer Cell Death” Amplifier to Boost Systemic Tumor Elimination. *Advanced Functional Materials* **2020**, *30* (22), 1909745. DOI: <https://doi.org/10.1002/adfm.201909745>.

(11) Aquib, M.; Yang, W.; Yu, L.; Kannaujiya, V. K.; Zhang, Y.; Li, P.; Whittaker, A.; Fu, C.; Boyer, C. Effect of cyclic topology versus linear terpolymers on antibacterial activity and biocompatibility: antimicrobial peptide avatars. *Chemical Science* **2024**, 10.1039/D4SC05797J. DOI: 10.1039/D4SC05797J.

(12) Pham, P.; Oliver, S.; Nguyen, D. T.; Boyer, C. Effect of Cationic Groups on the Selectivity of Ternary Antimicrobial Polymers. *Macromolecular Rapid Communications* **2022**, *43* (21), 2200377. DOI: <https://doi.org/10.1002/marc.202200377>.

(13) Namivandi-Zangeneh, R.; Kwan, R. J.; Nguyen, T.-K.; Yeow, J.; Byrne, F. L.; Oehlers, S. H.; Wong, E. H. H.; Boyer, C. The effects of polymer topology and chain length on the antimicrobial activity and hemocompatibility of amphiphilic ternary copolymers. *Polymer Chemistry* **2018**, *9* (13), 1735-1744, 10.1039/C7PY01069A. DOI: 10.1039/C7PY01069A.
